# Impact of Statin Therapy on Diabetes Incidence: Implications for Primary Prevention

**DOI:** 10.1007/s11886-024-02141-3

**Published:** 2024-09-20

**Authors:** Rishi Rikhi, Michael D. Shapiro

**Affiliations:** Center for Prevention of Cardiovascular Disease, Section on Cardiovascular Medicine, Department of Internal Medicine, Wake Forest University School of Medicine, Winston-Salem, NC 27157 USA

**Keywords:** Statin, Diabetes, Primary prevention, Atherosclerosis, Cardiovascular disease

## Abstract

**Purpose of Review:**

The present review aims to summarize current evidence, explore underlying mechanisms, and help guide clinicians regarding statin therapy and diabetes risk in primary prevention.

**Recent Findings:**

The observational and genetic epidemiology, as well as evidence from randomized controlled trials and meta-analyses, illustrate a modest, dose-dependent increase in risk of diabetes from statin therapy.

**Summary:**

Risk of new onset diabetes from statins appears to be greatest in those near the diagnostic threshold for diabetes or with diabetes risk factors prior to statin initiation. The risk of incident diabetes is vastly offset by the cardiovascular protection offered from statin therapy and should not deter guideline recommended statin initiation in primary prevention.

## Introduction

Beyond therapeutic lifestyle changes, statins are the cornerstone for lipid lowering therapy and global cardiovascular risk reduction in primary prevention [1]. In fact, statin therapy in primary prevention has been shown to reduce all-cause and cardiovascular mortality [2]. The number needed to treat to prevent 1 atherosclerotic cardiovascular disease (ASCVD) event with high intensity statin therapy in primary prevention over the course of 10 years is approximately 18 to 21 [3]. With multiple lines of evidence illustrating the cardiovascular benefit from statin therapy, 5 major organizations have provided clear recommendations for statin initiation in primary prevention [3].

In particular, the 2018 American College of Cardiology/American Heart Association Cholesterol Guideline provides specific recommendations for statin therapy in primary prevention, as follows:

1. Individuals with low-density lipoprotein (LDL)-cholesterol (LDL-C) ≥ 190 mg/dL: High-intensity statin therapy is indicated.
2. Individuals with diabetes aged 40–75 years with LDL-C between 70 and 189 mg/dL: Moderate-intensity statins are recommended, and high-intensity statins should be considered based on diabetes- specific risk enhancers, including long duration (≥ 10 years for type 2 diabetes or ≥ 20 years for type 1 diabetes), albuminuria, estimated glomerular filtration rate < 60mL/min/1.73m^2^, retinopathy, neuropathy, or ankle brachial index (ABI) < 0.9.
3. Individuals aged 40–75 years without diabetes and with an estimated 10-year ASCVD risk of ≥ 7.5% [calculated using the Pooled Cohort Equations (PCE)]: Moderate- to high-intensity statin therapy is recommended.
4. Individuals aged 40–75 years with an estimated 10-year ASCVD risk of 5-7.5%: Statin therapy should be considered, particularly if risk-enhancing factors are present [1].

Risk enhancers that can further refine risk include: South Asian ancestry, family history of premature ASCVD, chronic kidney disease, chronic inflammatory diseases, metabolic syndrome, premature menopause, pregnancy conditions, primary hypercholesteremia/hypertriglyceridemia, abnormal ABI, or elevation of specific biomarkers: lipoprotein(a), apolipoprotein B100, and high-sensitivity C-reactive protein [1]. The guidelines also address the use of coronary artery calcium testing to guide therapeutic decisions if uncertainty remains regarding statin initiation. A CAC score ≥ 100 Agatston units or ≥ 75th percentile for age/sex support statin initiation [1].

Statins reduce plasma LDL-C through competitive inhibition of 3-hydroxy-3-methylglutaryl-coenzyme A reductase (HMGCR), the rate limiting step in the cholesterol synthesis pathway. The decrease in hepatic cholesterol production leads to increased expression of hepatic surface LDL receptors (LDLR), and thus, increased plasma clearance of LDL [4]. The main cardiovascular benefit from statins relates to greater hepatic clearance of LDL and thus decreased circulating LDL. In primary prevention, every 1mmol/L reduction in LDL-C is associated with an approximately 23–24% reduction in major vascular events [5]. Other cardiovascular benefits from statins independent of LDL-C reduction have been suggested, such as plaque stabilization and anti-inflammatory effects. Despite these benefits, statin discontinuation rates remain high, largely due to statin associated side effects, with statin-associated muscle symptoms being the most common [4]. One risk that has garnered significant attention is the association between statin therapy and new-onset diabetes. This adverse effect is particularly concerning because individuals with diabetes have a 2 to 4 times higher risk of cardiovascular disease [4, 6]. In fact, in 2012, the United States Food and Drug Administration updated the statin safety label to include a statement that statins have been found to increase fasting serum glucose levels and glycosylated hemoglobin [7].

This review aims to synthesize current evidence on the association between statin use and incident diabetes, explore the underlying mechanisms, and discuss the implications for clinical practice in primary prevention.

### Observational Cohort Studies

Numerous cohort studies have yielded observational evidence linking statin use to the development of new-onset diabetes. In a study from the Women’s Health Initiative, which included 120,173 participants free of cardiovascular disease and diabetes, baseline statin use was associated with an almost 50% increased risk of new onset diabetes over a follow up of 1,004,466 person-years (HR 1.48; 95% CI 1.36–1.62). Findings were consistent across statin types, potency, and duration. The key limitations of this analysis were the inclusion of only female participants and a predominance of White participants, limiting generalizability. Additionally, diabetes was ascertained by self-report, which may have led to misclassification [8].

Compared to the data from the Women’s Health Initiative, a retrospective cohort study from Ontario, Canada found a much more modest association between statin therapy and diabetes in 227,994 participants free of cardiovascular disease. Carter et al. conducted a study comparing the risk of developing diabetes among users of various statins, using pravastatin as an active comparator as it has been shown to improve gluconeogenesis by raising levels of adiponectin in animal models. Atorvastatin (HR 1.20; 95% CI 1.10–1.30), rosuvastatin (HR 1.12; 95% CI 1.02–1.23), and simvastatin (HR 1.12; 95% CI 1.02–1.23) were associated with higher risk of new-onset diabetes. Conversely, fluvastatin (HR 0.98; 95% CI 0.79–1.22) and lovastatin (HR 1.01; 95% CI 0.82–1.23) were not associated with incident diabetes. These data suggest that the risk of incident diabetes and statin therapy may not be a class effect [9].

A study from the National Health Insurance Examinees further advanced the understanding of the link between statin use and diabetes by considering both the duration and dose of therapy. The study included individuals aged 40 and older with hypercholesterolemia, who had no prior history of ASCVD or diabetes. It followed 518,491 propensity-matched pairs for an average of 3.9 years. The findings revealed that statin users had nearly a 90% increased risk of developing new-onset diabetes (HR 1.88; 95% CI 1.85–1.93). Additionally, risk of diabetes increased with duration and intensity of statin treatment [10]. Collectively, these studies offer observational evidence of clear association between statin use and the incidence of diabetes.

### Genetic Studies

Advances in genomics have enabled the use of genetic instruments to demonstrate a likely causal relationship between statin use and incident diabetes. In a study by Swerdlow et al., two single nucleotide polymorphisms (SNPs) of HMGCR, rs17238484 and rs12916, were used as genetic proxies for statin inhibition of HMGCR. The study included a total of 223,463 participants from 43 observational studies. Per allele, both SNPs, rs17238484 and rs12916, were associated with greater risk of new-onset diabetes (OR 1.02; 95% CI 1.00-1.05) and (OR 1.06; 95% CI 1.03–1.09), respectively. Additionally, rs17238484 was associated with lower LDL-C, as well as increases in weight, waist circumference, insulin levels, and glucose levels. This study sheds light that HMGCR inhibition may be responsible for diabetes risk and related cardiometabolic abnormalities. However, it is not clear if this pathway is independent of LDLR upregulation and/or LDL-C reduction [11].

In another study using genetic instruments, *HMGCR* variants were used to construct genetic risk scores (GRS) that mimicked the effect of statins. A total of 112,772 individuals were included from 14 observational statin trials. The mean age of participants was 59.9 years, and the average LDL-C was 129.9 mg/dL. Participants were divided into two groups based on their genetic risk score (GRS): those above and those below the median, as well as into quartiles to investigate the dose-response relationship. Individuals above the median GRS had an LDL-C level that was 3.2 mg/dL lower and a 6.6% lower risk of myocardial infarction or death from coronary heart disease compared to those below the median GRS (OR 0.93; 95% CI 0.90–0.97). For a standardized difference of 10 mg/dL in LDL-C, the risk of myocardial infarction or death from coronary heart disease was 19.1% lower in individuals with HMGCR variants. Those with a GRS above the median had a greater risk of new-onset diabetes (OR 1.05; 95% CI 1.03–1.08). Additionally, a dose-response relationship was observed, with individuals in the highest quartile exhibiting the greatest risk of incident diabetes (OR 1.07; 95% CI 1.00-1.14). For a 10 mg/dL difference in LDL-C, the risk of new-onset diabetes was 12.7% higher (OR 1.13; 95% CI 1.06–1.20). Interestingly, GRS was not associated with baseline fasting glucose levels. However, the authors found that GRS was associated with incident diabetes in individuals with impaired fasting glucose, but not in those with normal fasting glucose at baseline. For every 10 mg/dL decrement in LDL-C, the risk of new-onset diabetes was increased in those with impaired fasting glucose (OR 1.19; 95% CI 1.00-1.41), but not in those with normal fasting glucose (OR 1.04; 95% CI 0.89–1.22). In this study, HMGCR GRS was associated with a dose-response increase in new-onset diabetes, but only in those with impaired baseline fasting glucose. Despite this increase in new-onset diabetes, the cardiovascular benefits significantly outweighed the risks [12].

### Randomized Controlled Trials

Adding to the observational evidence from cohort studies are data from randomized controlled trials, providing further insight on the association between statin use and incident diabetes. The Justification for Use of Statins in Prevention: an Intervention Trial Evaluating Rosuvastatin (JUPITER) was a randomized, double-blind, placebo-controlled trial investigating the cardiovascular benefit of rosuvastatin in primary prevention. A total of 17,802 participants with an LDL-C < 130 mg/dL and high sensitivity C-reactive protein ≥ 2 mg/L were randomized to 20 mg of rosuvastatin or placebo. The primary endpoint was a composite of non-fatal myocardial infarction, stroke, unstable angina hospitalization, arterial revascularization, or death due to cardiovascular disease. Participants with a history of ASCVD or diabetes were excluded, and incident diabetes was a pre-specified secondary aim of the study. Over a median follow up of 1.9 years, physician reported new-onset diabetes was higher in the rosuvastatin group compared to placebo (3.0% vs. 2.4%) [13]. In a secondary analysis, participants were divided into two groups based on the presence of one or more diabetes risk factors: hemoglobin A1c > 6%, body mass index ≥ 30 kg/m^2^, impaired fasting glucose, and metabolic syndrome. A total of 6,095 participants did not have any diabetes risk factors, and in this group, statin use was not associated with new-onset diabetes (HR 0.99; 95% CI 0.45–2.21). Conversely, in the 11,508 participants who did have at least one diabetes risk factor, statin use was significantly associated with incident diabetes (HR 1.28; 95% CI 1.07–1.54). In those with and without diabetes risk factors, rosuvastatin was found to reduce risk of the primary endpoint (HR 0.61; 95% CI 0.47–0.79) and (HR 0.48; 95% CI 0.33–0.68), respectively. Data from JUPITER illustrates the profound cardiovascular benefit from statin therapy compared to the relatively modest increase in incident diabetes. Additionally, new-onset diabetes was only present in those with at least one major risk factor for diabetes at baseline, suggesting statins may promote glycemic changes or insulin resistance, rather than cause de novo diabetes. Of note, the median follow up time in JUPITER was only 2 years, which may underestimate diabetes incidence when considering long term statin therapy in primary prevention [7].

Compared to the results from JUPITER, data from the West of Scotland Coronary Prevention Study Group (WOSCOPS) found decreased risk in new-onset diabetes. In this study, 6,595 men aged 45–64 with hypercholesterolemia and no prior ASCVD were randomized to receive either pravastatin 40 mg or a placebo. Over a mean follow up of 4.9 years, there was a 31% risk reduction of nonfatal myocardial infarction or death from coronary heart disease in the pravastatin group compared to placebo [14]. In a secondary analysis that included 5,974 men who had at least 2 post randomization glucose measurements, randomization to pravastatin was associated with a 30% lower risk of developing diabetes (HR 0.70; 0.50–0.99) [15]. Similar to the observational analysis by Carter et al., these findings suggest that pravastatin may have unique properties that facilitate improved glycemic control [9, 15]. Of note, new-onset diabetes in this study was defined as a fasting glucose ≥ 126 mg/dL, over two readings, with at least one reading ≥ 36 mg/dL above the baseline measurement. Thus, the more stringent criteria for dysglcycemia in this study may have underestimated new cases of diabetes [15].

### Meta-Analyses

Over the last 15 years, several meta-analyses have provided estimates on the effect size between statin use and new-onset diabetes. In a meta-analysis by Rajpathak et al., 57,593 individuals from 6 different randomized controlled trials were included and followed for a weighted mean of 3.9 years. Various statins, including pravastatin, rosuvastatin, simvastatin, and atorvastatin, were compared to placebo and found to reduce the risk of cardiovascular disease by 8–44%. The authors conducted two analyses: the first included five statin trials excluding WOSCOPS, and the second included all six trials. In the first analysis, statin therapy was associated with a modest increase in new-onset diabetes (RR 1.13; 95% CI 1.03–1.24). However, in the second analysis, statin therapy did not significantly increase the risk of diabetes (RR 1.06; 95% CI 0.93–1.23). While no significant heterogeneity was found in the first analysis, significant heterogeneity was observed in the second analysis. In a meta-regression analysis, age and female sex were both significantly associated with incident diabetes from statins. Therefore, data from WOSCOPS may be an outlier given the inclusion of only men, as well as having a higher threshold for diagnosing diabetes. Focusing on the initial analysis of 5 statin trials, the meta-analysis by Rajpathak et al. illustrates a small increase in new-onset diabetes from statin therapy [16].

A similar modest increase in new-onset diabetes was seen in a larger meta-analysis by Sattar et al. that included 91,140 participants without baseline diabetes from 13 different statin trials. Over the course of 4 years, incident diabetes was diagnosed in 2,226 participants assigned to statin therapy and in 2,052 participants assigned to placebo. Thus, those treated with statin therapy had higher risk of new-onset diabetes (OR 1.09; 95% CI 1.02–1.17). A subgroup analysis demonstrated a similar risk of diabetes between lipophilic and hydrophilic statins (OR 1.10; 95% 0.99–1.22) and (OR 1.08; 95% CI 0.98–1.20), respectively. There was little heterogeneity between trials and risk of diabetes appeared to be strongest in trials with older participants [17].

Preiss et al. compared high- versus moderate-dose statin therapy. This meta-analysis included 37,752 participants without baseline diabetes from 5 statin trials. Incident diabetes was diagnosed in 1,449 participants randomized to high-dose statin therapy and 1,300 participants assigned to moderate-dose statin therapy over a weighted mean follow up of 4.9 years. Thus, there was an increase in incident diabetes in those assigned to high-dose statin therapy compared to moderate-dose statin therapy (OR 1.12; 95% 1.04–1.22). The authors found lower risk of cardiovascular disease in those treated with high- versus moderate-dose statin therapy (OR 0.84; 95% 0.75–0.94). In summary, the number needed to harm for incident diabetes was 498 and the number needed to treat to prevent cardiovascular disease was 155 [18].

Recently, a meta-analysis of statin trials analyzed patient-level data to clarify the link between statin use and new-onset diabetes, while also identifying specific risk factors for developing diabetes. A total of 78,445 participants from 14 trials that investigated low- or moderate-intensity statin therapy compared to placebo were included. In this study, low- or moderate-intensity statins were associated with a higher risk of incident diabetes, with 2,420 new cases (1.3% per year) in the statin group compared to 2,214 new cases (1.2% per year) in the placebo group (RR 1.10; 95% CI 1.04–1.16). The authors also included 19,794 participants from 2 trials investigating high intensity statin therapy compared to placebo and found a 36% increase in diabetes risk from high intensity statins (RR 1.36; 95% CI 1.25–1.48). Using 4 trials comparing intensity of statin therapy, the authors found that higher intensity statins lead to greater risk of incident diabetes (RR 1.10; 95% CI 1.02–1.18). While there was a significant risk of diabetes associated with statin use, the authors found that the mean increase in glucose secondary to statins vs. placebo was only 0.04 mmol. Additionally, 62% of new-onset diabetes cases occurred in individuals in the highest quartile of baseline glycemia. This suggests that statins may cause dose-dependent increases in glycemia, leading to a higher risk of new-onset diabetes, especially in those who are near the biochemical diagnostic threshold for diabetes at baseline [19].

### Mechanisms

There is limited evidence on the mechanisms linking statins to new-onset diabetes. Some theories suggest that weight gain associated with statin use may contribute to the increased incidence of diabetes. A study using HMGCR genetic instruments found that the association with higher glucose and insulin concentrations was attenuated when adjusted for body mass index. However, weight gain is unlikely to be the primary mechanism, as the extent of weight gain was insufficient to fully explain the increased diabetes risk. For instance, a meta-analysis of 129,170 participants showed an average body weight increase of only 0.24 kg over a mean duration of 4.2 years. Furthermore, in this same analysis, while both placebo- or standard care- controlled trials, as well as high- versus moderate dose trials was associated with higher risk of new-onset statin associated diabetes, weight gain was only seen in the former [11]. Lastly, although not confirmed in randomized controlled trials, genomic data suggests that PCSK9 inhibitors (PCSK9i) lead to greater risk of diabetes, but not weight gain [12]. Given these findings, a common mechanistic pathway likely exists between statins and PCSK9i.

A study using genetic instruments of *PCSK9*, *HMGCR*, and *LDLR* found each of them to have almost an identical increase in new-onset diabetes [12]. Thus, the increase in incidence diabetes from statin therapy may be due to greater expression of LDLR. Inhibition of HMGCR by statins leads to greater expression of LDLR not only at the hepatic surface, but also at the surface of the pancreas. The higher surface expression of LDLR could lead to accumulation of lipid toxic compounds in the pancreas. These compounds could then damage beta cells and impair insulin secretion. Further supporting this theory is data from individuals with familial hypercholesterolemia who have fewer functioning LDLR and have less risk of diabetes. Several other mechanisms are being studied, however, almost all data are in vitro. Clinical studies and randomized trials are needed to provide a mechanistic understanding behind statin use and incident diabetes [20].

## Conclusion

Multiple lines of evidence have shown a modest, dose dependent increase in new-onset diabetes from statin use. The risk is predominately in individuals who are near the diabetes diagnostic threshold prior to starting statins or have diabetes risk factors [13, 19]. While guideline screening for diabetes should occur in primary prevention, there is not enough data to support routine monitoring of glycemic indices solely due to statin initiation. Additionally, the risk of incident diabetes is vastly offset by the cardiovascular protection offered from statin therapy [12]. Future randomized controlled trials should use specific predefined diabetes outcomes, assess for microvascular complications, and include measures of insulin resistance and production to further explore mechanisms. In conclusion, risk of incident diabetes should not deter guideline recommend statin initiation in primary prevention.

## Key References

Cholesterol Treatment Trialists’ Collaboration. Electronic address cnoau, Cholesterol Treatment Trialists C. Effects of statin therapy on diagnoses of new-onset diabetes and worsening glycaemia in large-scale randomised blinded statin trials: an individual participant data meta-analysis. Lancet Diabetes Endocrinol 2024; 12:306–319.

**Findings from this Recent meta-analysis Using Participant Level data Demonstrate a Small dose Dependent Increase in Diabetes from Statin Therapy.**

Andersson NW, Corn G, Dohlmann TL et al. LDL-C Reduction With Lipid-Lowering Therapy for Primary Prevention of Major Vascular Events Among Older Individuals. J Am Coll Cardiol 2023; 82:1381–1391.

**Findings from this study highlight the cardiovascular benefit from statin therapy in primary prevention.**

## Data Availability

No datasets were generated or analysed during the current study.
